# Stabilizing versus Destabilizing the Microtubules: A Double-Edge Sword for an Effective Cancer Treatment Option?

**DOI:** 10.1155/2015/690916

**Published:** 2015-09-21

**Authors:** Daniele Fanale, Giuseppe Bronte, Francesco Passiglia, Valentina Calò, Marta Castiglia, Florinda Di Piazza, Nadia Barraco, Antonina Cangemi, Maria Teresa Catarella, Lavinia Insalaco, Angela Listì, Rossella Maragliano, Daniela Massihnia, Alessandro Perez, Francesca Toia, Giuseppe Cicero, Viviana Bazan

**Affiliations:** ^1^Department of Surgical, Oncological and Oral Sciences, Section of Medical Oncology, University of Palermo, 90127 Palermo, Italy; ^2^Department of Surgical, Oncological and Oral Sciences, Section of Plastic Surgery, University of Palermo, 90127 Palermo, Italy

## Abstract

Microtubules are dynamic and structural cellular components involved in several cell functions, including cell shape, motility, and intracellular trafficking. In proliferating cells, they are essential components in the division process through the formation of the mitotic spindle. As a result of these functions, tubulin and microtubules are targets for anticancer agents. Microtubule-targeting agents can be divided into two groups: microtubule-stabilizing, and microtubule-destabilizing agents. The former bind to the tubulin polymer and stabilize microtubules, while the latter bind to the tubulin dimers and destabilize microtubules. Alteration of tubulin-microtubule equilibrium determines the disruption of the mitotic spindle, halting the cell cycle at the metaphase-anaphase transition and, eventually, resulting in cell death. Clinical application of earlier microtubule inhibitors, however, unfortunately showed several limits, such as neurological and bone marrow toxicity and the emergence of drug-resistant tumor cells. Here we review several natural and synthetic microtubule-targeting agents, which showed antitumor activity and increased efficacy in comparison to traditional drugs in various preclinical and clinical studies. Cryptophycins, combretastatins, ombrabulin, soblidotin, D-24851, epothilones and discodermolide were used in clinical trials. Some of them showed antiangiogenic and antivascular activity and others showed the ability to overcome multidrug resistance, supporting their possible use in chemotherapy.

## 1. Introduction

Microtubules are dynamic and structural cellular components, typically formed by 13 protofilaments, which constitute the wall of a tube; each of the protofilaments consists of a head-to-tail arrangement of *α*/*β* tubulin heterodimers [1]. They are involved in several cell functions, including cell shape, motility, and intracellular trafficking. In proliferating cells, they are one of the essential components in the division process through the formation of the mitotic spindle. This event can take place because of the dynamic nature of microtubules through polymerization and depolymerization cycles [2]. As a result of these functions, tubulin and microtubules are targets for anticancer agents [3, 4]. Microtubule-targeting agents can be divided into two groups: microtubule-stabilizing and microtubule-destabilizing agents. The former bind to the tubulin polymer and stabilize microtubules, while the latter bind to the tubulin dimers and destabilize microtubules [5, 6].

**Figure 1:**
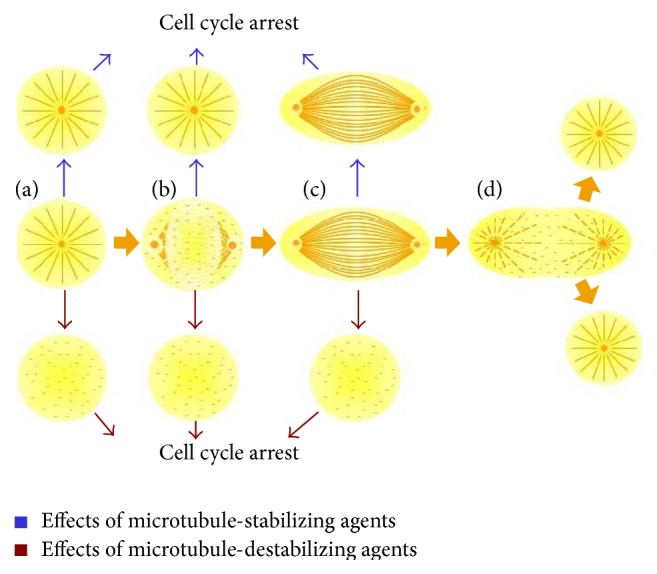
The dynamic nature of cytoskeleton is due to cycles of microtubule catastrophes. (a) Model structure of assembled cytoskeleton. The variety of shapes and sizes of the microtubule cytoskeleton is as great as the number of different cell types. In interphase, microtubules are long and stable because there are almost no catastrophes. (b) In mitosis, catastrophes are relatively frequent, resulting in highly dynamic microtubules that reach a steady-state length after a few minutes of growth (c). (d) After the segregation of chromatids, a new cycle of depolymerization and polymerization begins, resulting in a new stable microtubule cytoskeleton in daughter's cells (d). Blue and red arrows indicate effects of stabilizing and destabilizing agents, all resulting in cell cycle arrest.

Despite these differences, alteration of tubulin-microtubule equilibrium leads to the same final result: it disrupts the mitotic spindle, halting the cell cycle at the metaphase-anaphase transition and eventually resulting in cell death [7] (Figure 1).

Clinical application, however, has unfortunately shown several limits, such as a high level of neurological and bone marrow toxicity and the emergence of drug-resistant tumor cells due to the overproduction of P-glycoprotein (Pgp), an ATP-binding cassette (ABC) transmembrane transporter [8], the overexpression of different beta-tubulin isotypes, including *β*III-tubulin [9, 10], or tubulin mutations [11].

Several natural and synthetic microtubule-targeting agents, exhibiting antitumor activity and increased efficacy in comparison to traditional drugs in various preclinical and clinical studies, have been discovered and their mechanisms have been elucidated [12, 13]. Apart from the well-known antimitotic function, for some of these drugs antiangiogenic and antivascular activity were demonstrated; for others the ability to overcome multidrug resistance was found. Many of these new generation microtubule-targeting agents are still under evaluation for clinical use. Some of them showed good tolerability and antitumor activity in particular cancers.

This review provides an overview of those microtubule-targeting drugs which are to date under clinical evaluation. A particular attention will be paid to the translation of preclinical data into the design of clinical trials.

## 2. Microtubule-Destabilizing Agents

Colchicine and Vinca alkaloids are two of the first microtubule-destabilizing agents to be discovered. These two compounds depolymerize microtubules by interacting with various *β*-tubulin sites. In particular, Vinca alkaloids interact with tubulin at specific binding sites which differ from those of other agents, including colchicine or taxanes, interfering with microtubule dynamics, blocking polymerization at the end of the mitotic spindle, and leading to metaphase arrest. Thanks to their peculiar mechanism of action, Vinca alkaloids have been widely used in anticancer therapy, usually in combination with other chemotherapeutic agents which do not have cross-resistance with them. First-generation Vinca alkaloids such as vinblastine have been included in the treatment protocol of both Hodgkin and non-Hodgkin lymphomas and testicular carcinoma, while vincristine has been approved for several years in the treatment of hematological tumors such as acute leukemia and multiple myeloma but also of rare tumors such as rhabdomyosarcoma and neuroblastoma. However, vincristine treatment was associated with a severe neurotoxicity, while the suppression of the bone marrow was more frequently reported during vinblastine therapy [14]. Second-generation semisynthetic Vinca alkaloids, vinorelbine and vindesine, have shown a broader spectrum of antitumor activity* in vitro*, along with a decreased neurotoxicity. Vinorelbine was approved as single agent and in combination therapy for the treatment of both hematological and solid tumors, including lung cancer, breast cancer, and gynecological tumors [15]. Recently, another synthetic Vinca alkaloid, vinflunine, has been approved in Europe for the second-line treatment of metastatic urothelial carcinoma. It is the first fluorinated microtubule inhibitor, which was associated with a higher antitumor activity than other Vinca alkaloids, showing also an excellent safety profile [16].

In order to overcome the clinical limits of these agents, in the last years attention has been focused on natural and synthetic compounds with a different structure but which act in a similar way [7, 17] (Table 1).

### 2.1. Cryptophycins

**Figure 2:**
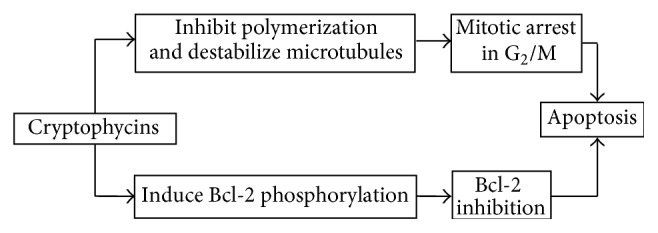
Mechanism of action of cryptophycins.

**Figure 3:**
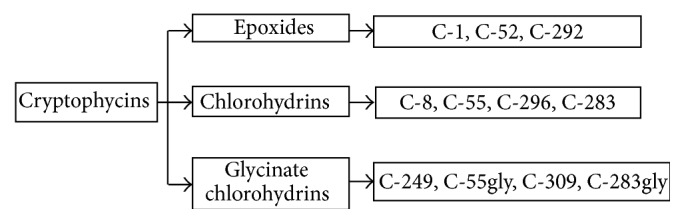
Classification of cryptophycins.

Cryptophycins are synthetic derivatives of macrocyclic depsipeptides, isolated by* Nostoc sp.* [18]. They block cell division and prevent the correct formation of the mitotic spindle, by inhibiting tubulin polymerization, probably at the binding site of the* Vinca alkaloids* [19]. In particular, C-52 and C-55 induce apoptosis by means of Bcl-2 hyperphosphorylation and inactivation [20–22] (Figure 2). These compounds are able to induce this phosphorylation at a greater extent than other microtubule inhibitors [23]. The first form discovered was epoxide cryptophycin 1, which showed antitumoral activity both in preclinical* in vitro* (colon, breast, ovarian, lung, and nasopharyngeal carcinomas) and* in vivo* (lung, breast, and prostate tumors) models. This has led to isolation and synthesis of cryptophycin analogs, divided into epoxides, chlorohydrins, and glycinate chlorohydrins [24] (Figure 3).

Cryptophycin 8 is the first C-1 analog synthesized in order to improve its antitumoral efficacy by means of conversion of the epoxide group into chlorohydrin. Its activity has been shown both in murine and human tumors. Although it is not as powerful as C-1, it is more soluble in water and has a stronger therapeutic effect. Nevertheless, it is still too unstable in solution to be considered clinically relevant [25].

#### 2.1.1. Cryptophycins 52 and 55

Cryptophycin 52 (LY355703) is a synthetic epoxide, used in phase II clinical trials, which presents a cytotoxic effect 400 times stronger than paclitaxel and* Vinca* alkaloids [26, 27]. It shows* in vitro* antitubulin, antimitotic, and cytotoxic activity which is dose-dependent against tumor cells. Furthermore, its activity has been evaluated both in murine tumor models and in human tumor xenografts [23]. C-52 resulted to be also effective against multidrug-resistant tumors [26, 28, 29].

Paclitaxel and the Vinca alkaloids are sensitive to the multidrug resistance (MDR) transporters P-glycoprotein (P-gp, MDR-1) and/or MDR-associated protein (MRP-1). Cryptophycin 52 was tested for its sensitivity to multidrug resistance in several paired cell lines in which a sensitive parental line was matched with a multidrug-resistant derivative line. Compared to other antimitotic agents (paclitaxel, vinblastine, and vincristine), the potency of cryptophycin 52 was shown to be minimally affected in multidrug-resistant cells compared to their sensitive parental lines [30]. Cryptophycin 52 fragment A analogues was synthesized to improve the potency and the aqueous solubility of the molecule allowing for the modification of its formulation. However, the same functional groups that rendered these analogues more potent and more water soluble also contributed to making them better substrates of the Pgp efflux pump. It is an unacceptable feature in the development of a clinically relevant antitumor agent [29].

Preclinical toxicological studies on animals (rats and dogs) have shown that above a certain concentration level C-52 causes secondary effects such as neutropenia and gastrointestinal problems but not neurotoxicity. These studies have allowed evaluating the optimum phase II dosage and tracing the plasma pharmacokinetic profile [26]. Furthermore, phase I clinical trials identified 1.5 mg/m^2^ as a well-tolerated dose level of C-52. It was delivered as a 2-hour i.v. infusion on day 1 and day 8 repeated every 3 weeks [31]. This schedule was employed in a phase II study to determine the activity of C-52 in non-small cell lung cancer (NSCLC) patients previously treated with platinum-based chemotherapy and to characterize its toxicity profile. A good rate of disease stabilization and an unacceptable toxicity was found in this setting [32]. Also, a multicenter trial was performed to evaluate the same schedule of the drug in patients with platinum-resistant advanced ovarian cancer. A considerable clinical benefit without serious adverse events was achieved [28]. Afterwards, these phase II clinical trials were terminated due to significant neurological toxicity [12].

Cryptophycin 55, a C-52 chlorohydrin, shows higher cytotoxic activity and therapeutic efficacy than its epoxide precursor, but its low stability in solution has delayed its clinical application [33]. This problem has been overcome, however, by means of the synthesis of glycinate esters (C-55gly, C-283gly, and C-309) which show not only an* in vivo* activity similar to their precursors but also a high level of stability [34].

Treatment with C-52 and C-55 combined with other chemotherapy agents has produced synergic effects without increased toxicity, bringing about a greater survival rate in ovarian carcinoma murine models [23, 28]. The use of human tumor xenografts has made it possible to evaluate C-52 and C-55 activity combined with cisplatin, carboplatin, and oxaliplatin in different tumors. C-52 showed a synergic effect only when associated with cisplatin, whereas C-55 showed increased activity with all the platinum compounds [35].* In vivo* antitumoral activity of C-52 and C-55 has been assessed in combination with radiotherapy (2*γ*) or with 5-FU in tumor xenografts, showing an increased effect. Pharmacokinetic analyses performed in murine models have demonstrated that C-52 concentration in the tumor increased after administration and remained high for 24 hours. The mean life of C-55 was the longest in the liver, intermediate in tumor tissue, and less in plasma. After C-55 administration, the mean life of C-52 was the longest in tumor tissue, less in plasma, and even less in the liver, suggesting almost total conversion of C-55 into C-52 in the tumor. The greater C-52 accumulation in tumor tissue depends on the bioconversion of C-55 in C-52 and different binding affinities towards different tissue proteins. The use of C-55 to deliver C-52 increased the retention of C-52 in tumor tissue and reduced its presence in all studied normal tissues. Furthermore, extracellular acid pH of the tumor increased C-55 stability, whereas intracellular basic pH encouraged bioconversion by stimulating its pharmacological activity [36].

The obtained results indicated that C-52 and C-55 fulfilled all the criteria required by ideal chemotherapy agents, since they showed an action mechanism against a specific target and considerable activity against drug-resistant cells. However, the lack of response observed in some tumors and peripheral neuropathy have been limiting factors in the development of these agents leading to termination of their study.

#### 2.1.2. Second-Generation Cryptophycins

C-309, C-249, and C-283 are second-generation candidates for clinical use. The first two are glycinate esters, synthesized in order to provide a higher chemical stability and more solubility in water. C-309 is a derivative of C-296 which has proved to have more therapeutic activity than C-55, C-283, C-249, and C-296; it is able to bring about a complete or partial regression of murine tumors at lower doses than those of other glycinate analogs. C-249 derives from C-8 and is active against MDR tumors. Moreover, it has the advantage of being easier to synthesize.

These second-generation analogs have proved to be up to 1000 times as active as those of the first clinical candidates (C-52) but with the same or even less toxicity [34].

### 2.2. Combretastatins

The combretastatins, isolated from* Combretum caffrum*, are molecules structurally related to colchicine which have been extensively developed since the late 1990s as vascular-disrupting agents (VDAs) [37]. The vascular-disrupting effect of these compounds is present well below the maximum tolerated dose, with a wide therapeutic window [38]. A number of combretastatins are currently in clinical trials: combretastatins A4- and A1-phosphate, verubulin, crolibulin, plinabulin, and ombrabulin [12].

#### 2.2.1. CA-4-P

Combretastatin A-4 interacts with tubulin at the colchicine binding site but not in the same pseudoirreversible manner. It is used as a combretastatin A-4 3-O-phosphate (CA-4-P), a prodrug which is soluble in water and transformed into its active form by endogenous phosphatases [39]. It showed cytotoxicity in tumor cell lines and in human endothelial cells, HUVEC, which are sensitive to the drug only if they are actively proliferating, suggesting a potential use as an antiangiogenic agent [38]. By interfering with microtubule polymerization and with mitotic spindle assembly, CA-4-P induces G2/M arrest, thus bringing about cell death by either mitotic catastrophe or apoptosis [38, 40, 41].

Recent computational studies, using fluorescence spectroscopy, identified a potential binding site on *γ*-tubulin for both CA-4-P and colchicines [42]. Since high levels of *γ*-tubulin have been reported in poorly differentiated and aggressive brain tumors, such as human glioblastoma and medulloblastoma [43, 44] and lung [45] and breast cancer [46], the discovery of a potential site interaction on this molecule would offer the possibility of targeting inhibition with a new class of chemotherapeutic agents. However, the experimental validation of such interesting observation is underway.

CA-4-P (also known as Zybrestat or fosbretabulin) shows a potent* in vivo* antivascular activity since it causes a rapid and widespread reduction of the tumoral blood flow and an increased vascular resistance, effects which are extremely reduced in the normal tissues [47]. At a dose of 1/5–1/10 of the maximum tolerated dose (MTD), the central area of the tumor undergoes hemorrhagic necrosis, while a thin peripheral ring of live cells remains [38, 48, 49]. On the contrary, colchicine and other drugs act only at approximately MTD [50]. This constitutes an important advantage for the therapeutic application of CA-4-P. An immediate effect of CA-4-P treatment is an increased vascular permeability, which is important for the reduction of blood flow through vascular collapse, and an increased viscosity consequent to fluid loss from the vasculature. However, endothelial barrier function alterations and increased vascular permeability might contribute to hastening tumor cell extravasation, causing progression to stages of greater malignancy, with heightened invasiveness and, in some cases, increased distant metastasis. It is no coincidence that susceptibility of tumors to CA-4-P showed a positive correlation with tumor vascular permeability [51]. Experiments conducted on HUVEC cells have shown that the CA-4-P-inducted microtubule depolymerization triggers off the actin reorganization through Rho activation and MLC (Myosin Light Chain) phosphorylation, thus causing rounding and retraction of cells and membrane blebbing. These events are associated with increased permeability, while the morphological cell change might contribute to determining the effects observed* in vivo* by means of vascular constriction [52, 53]. Furthermore, since CA-4-P interferes with the formation of stress fibers, it inhibits the VE-cadherin/*β*-catenin complex, thus leading to the destabilization of cell-cell junctions and increasing endothelial permeability [54].

The* ex vivo* perfusion of animal tumors highlights a lower increase in vascular resistance to that found* in vivo*, suggesting that the blood might also contribute towards the antivascular action of the drug [48]. It has been demonstrated that CA-4-P induces increased expression of endothelial CAM, responsible for the observed neutrophil recruitment which* in vivo* probably contributes both to vascular damage and to tumor cell death [55].

**Figure 4:**
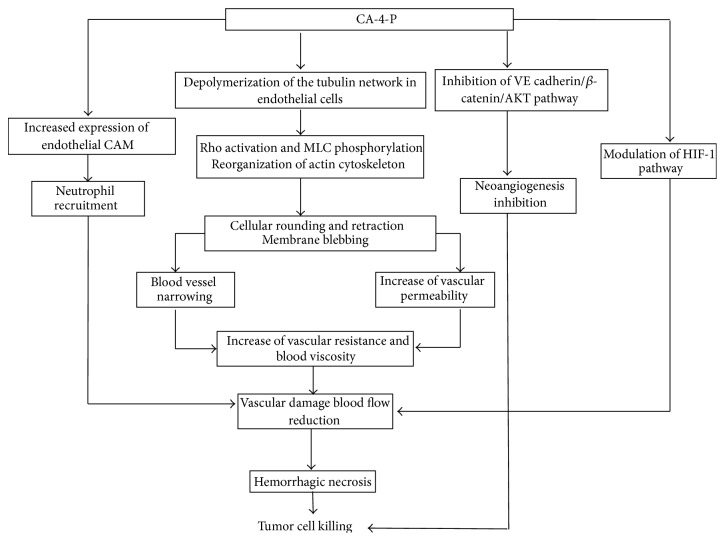
Combretastatin A-4-P: mechanisms of action at tumor level.

Apart from being an antivascular agent, CA-4-P inhibits the formation of new blood vessels, both* in vitro* and* in vivo*, presumably through inactivation of the VE-cadherin/*β*-catenin complex and Akt, all proteins required for cell adhesion, survival, and proliferation during neoangiogenesis. The same study has shown that smooth muscle cells, which are resistant to the drug, interfere with its antiangiogenic activity* in vitro*, suggesting that they may confer resistance to the endothelium by stabilizing cell-cell junctions [54]. CA-4-P selectivity towards the neoplastic tissue might therefore depend on the immaturity of tumor vessels, together with the proliferative status of tumor endothelial cells. Moreover, CA-4-P reduces* in vitro* HIF-1 expression (Hypoxia Inducible Factor-1) under hypoxia mainly in endothelial cells compared to that in cancer cell lines, suggesting a further possible mechanism of action for the drug [56] (Figure 4).

However, the effects of CA-4-P on tumor growth are not particularly significant, probably because of the persistent presence of vital peripheral cells [50], although the administration of several doses compared to the same total dose of the drug does increase its antitumoral effect [57, 58]. Furthermore, CA-4-P activity is directly proportional to tumor size [49]. This aspect, together with its capacity to act on the tumor core, differentiates this drug from more common therapeutic approaches, which target the peripheral tumor area. These complementary properties, together with the limited action of CA-4-P as a single agent, have led to experimentation involving combined treatments. It has been demonstrated that CA-4-P increases the response to radiotherapy and hyperthermia in treated tumors [57, 59] and, what is more, leads to a 90% increase in the retention of the anti-CEA antibody marked with I^131^ in the tumor, which is eradicated in 83% of the cases [60]. Similarly, CA-4-P increases the effect of chemotherapy drugs such as cisplatin, vinblastine, 5-fluorouracil, and irinotecan [57, 61].

The overall* in vivo* results obtained with CA-4-P have led to its introduction in phase I clinical trials [12, 62–66]. A phase I trial was performed to determine the MTD, safety, and pharmacokinetic profile of CA-4-P. This study showed absence of traditional cytotoxic side effects, with a toxicity profile which seems consistent with a “vascularly active” drug [67, 68]. The effects on tumor blood flow were assessed using dynamic contrast-enhanced magnetic resonance imaging (DCE-MRI) techniques. Dosages < or = 60 mg/m^2^, as a 10 min infusion at 3-week intervals, define the upper boundary of the MTD. Similar effects were seen in other phase I clinical trials using a weekly and daily schedule [69, 70]. Afterwards, a further phase I trial investigated the combination of CA-4-P with carboplatin. A greater thrombocytopenia was observed as a consequence of altered carboplatin pharmacokinetics [63].

In order to improve its efficiency and reduce its side effects, a specific therapeutic system has been realized, based on the use of liposomes containing CA-4-P, carrying superficial RGD-peptides able to bind with the *α*_v_ *β*3 integrins overexpressed on proliferating tumor endothelium.* In vitro* tests have demonstrated the specificity and stability of the system, essential properties for its* in vivo* application [71]. To date, phase II/III clinical trials in lung and thyroid cancer are currently being evaluated [12]. These studies showed that CA-4-P with or without carboplatin and paclitaxel combination therapy was well tolerated in thyroid cancer patients, although it did not meet statistical significance in OS improvement [72]. Instead, preliminary data suggests survival benefits and increased responses without significant additional toxicity in NSCLC patients treated with CA-4-P in combination with carboplatin, paclitaxel, and bevacizumab compared to patients treated with carboplatin, paclitaxel, and bevacizumab only [73].

#### 2.2.2. CA-1-P

Combretastatin A1 phosphate (also known as Oxi4503), a CA-1 water-soluble prodrug, shows a powerful antivascular activity. When used in murine and human tumor xenografts at much lower doses than those required by CA-4-P, CA-1-P brings about a drastic reduction of blood flow, with resulting necrosis [74]. CA-1-P causes an increase in vessel permeability, in VEGF production and apoptosis induction in endothelial cells [75]. At high doses it is more easily tolerated than CA-4-P and shows a much higher antitumoral activity, leading to complete regression of human tumors even at extremely low doses [74]. Excellent results have been obtained with combined treatments involving several chemotherapy agents [76].

*In vitro* pharmacokinetic studies have suggested that CA-1-P is transformed into a more reactive metabolite than CA-4-P, which is responsible for most of the antitumoral activity; this has formed the basis for further clinical developments of the drug as an antivascular and antitumor agent [77]. The drug has completed the phase I evaluation as a potential anticancer drug at three different centres in the United Kingdom, and it was studied in other phase I clinical trials [78, 79]. Recently, a new series of combretastatin derivatives have been synthesized and evaluated in seven cancer cell lines, exhibiting good anticancer activity [80, 81].

#### 2.2.3. Ombrabulin

Ombrabulin (also known as AC-7700) is a serinamide hydrochloride, synthetic derivative of CA-4-P, which inhibits growth in a large number of drug-resistant animal tumors and carcinogen-induced tumors [39, 82]. Differently from CA-4-P, it does not act directly on the tumor vessels but instead causes constriction of the arterioles, resulting in complete downstream arrest of the blood flow and tumor growth [83]. These effects are obtained at doses half of MTD and 100 times less than that of CA-4-P [84]. Finally, the combination of AC-770 with cisplatin increases the effect of both drugs in murine tumors, with curative effects, and in human tumor xenografts [85]. In 2002, AC-7700 was introduced into phase I clinical trials in the United States and in Europe (AVE8062,* Aventis Pharma*). Recently, ombrabulin in combination with cisplatin was used in a phase III clinical trial for patients with advanced soft-tissue sarcomas after failure of anthracycline and ifosfamide chemotherapy, significantly improving progression-free survival. However, this improvement was not clinically relevant, despite being statistically significant [86].

### 2.3. Dolastatins

Dolastatins are pseudopeptides isolated from the sea hare* Dolabella auricularia* [50]. Dolastatins 10 and 15 showed antiproliferative activity. These agents induce apoptosis through interaction with tubulin [87]. Dolastatin 10 is a natural peptide able to interfere in microtubule assembly by means of the noncompetitive binding to the Vinca alkaloid site [88]. A phase II trial investigated dolastatin 10 in NSCLC patients. A low response rate was observed, even though a good tolerability was achieved. Myelosuppression was confirmed as the only noteworthy toxicity [89].

Other phase II clinical trials of dolastatin 10 were carried out in patients with metastatic melanoma, advanced colorectal and breast cancers, recurrent platinum-sensitive ovarian carcinoma, and hormone-refractory metastatic prostate adenocarcinoma [90–94]. These studies confirmed the same results previously obtained in terms of tumor response and toxicity. No activity was found in advanced pancreaticobiliary cancers and metastatic soft-tissue sarcomas [95, 96]. For this reason, it was suggested to not pursue the clinical development of this drug in further studies, not only because of its side effects [97] but also because of the low mean survival rate of the treated patients [95].

#### 2.3.1. Soblidotin

Soblidotin (TZT-1027) is a synthetic analog of dolastatin 10 which inhibits the growth of several tumoral cell lines and induces caspase-3-dependent apoptosis. It shows* in vivo* antivascular effects in tumoral models overexpressing VEGF and in murine colon tumors, with an increase in vascular permeability, vessel closure, and widespread hemorrhage. Soblidotin also shows antitumoral activity in vincristine-, docetaxel-, and paclitaxel-resistant tumors, which makes it a potential chemotherapy drug for use in tumors which do not respond to other microtubule inhibitors [98].

The first two European phase I clinical trials identified a recommended dose of soblidotin between 2.4 and 2.7 mg/m^2^ for a 3-weekly administration with neutropenia, fatigue, and a reversible peripheral neuropathy as the DLT. Moreover neurological side effects seemed to correlate with previous exposure to other neurotoxic agents such as platinum compounds. No correlation was found with body surface area suggesting possible use of flat dose regimen for next trials [99, 100]. In a Japanese phase I clinical trial MTD of 1.5 mg/m^2^ administered on days 1 and 8 in 3-week courses was found [101]. A combination of this drug with carboplatin was also tested. The recommended dose was 1.5 mg/m^2^ for soblidotin and AUC 5 for carboplatin and no pharmacokinetics interaction was observed [102]. In NSCLC patients a phase I trial indicated a recommended dose of 4.8 mg/m^2^, administered every 3-4 weeks as recommended dose [103].

Phase II clinical investigations suggested activity in advanced or metastatic soft-tissue sarcomas with prior treatment with anthracycline-based chemotherapy. This study confirmed tolerability profile, but objective response was demonstrated in none of the patients [104]. Another phase II trial showed no anticancer activity for soblidotin in NSCLC patients previously treated with platinum-based chemotherapy [105].

#### 2.3.2. Dolastatin 15

Dolastatin 15 is very similar to dolastatin 10. It was demonstrated by chromatography that the binding domain is the same as Vinca alkaloids and antimicrotubule peptides. The site of the binding is not a well-defined locus but a series of overlapping domains [106]. This drug showed an effect on growth and differentiation in leukaemia cell lines [107], induction of apoptosis through Bcl-2 phosphorylation in small cell lung cancer cell lines [108], and G2/M cell cycle arrest in human myeloma cell lines [109]. Romidepsin (Istodax), a dolastatin 15 analog, which also possesses activity as a histone deacetylase inhibitor, was found to be active in cutaneous T-cell lymphoma with a 34% objective response rate and for this it was approved in 2009 [110, 111]. Other two analogues of dolastatin 15 are used in clinical trials: cemadotin and tasidotin.

#### 2.3.3. Cemadotin

Cemadotin (LU103793) exerts its effect by inhibition of microtubule polymerization [112]. This drug is not able to inhibit the binding of vinblastine to tubulin and it can suppress microtubule growth without a significant microtubule depolymerization [113]. This agent was first evaluated in three phase I clinical trials for advanced refractory solid tumors with different schedules. Daily 5-day every 3 weeks schedule identified a recommended dose of 2.5 mg/m^2^ per day. It was associated with neutropenia, peripheral edema, and liver function test abnormalities as DLTs. This dose showed lack of prohibitive cardiovascular effects. Acceptable general toxicity profile has allowed prompting phase II trials [114]. Meanwhile, cemadotin was studied for 24-hour intravenous (i.v.) continuous infusion every three weeks. 15 mg/m^2^ was the recommended dose for this schedule. Hypertension was highlighted as the DLT, even if its nature remained unclear [115]. Even 5-day continuous intravenous (CIV) infusion was investigated. MTD was 12.5 mg/m^2^. There were moderate nonhematologic toxicities and no evidence of the cardiovascular toxicity [116]. Pharmacokinetic analysis in these phase I trials suggested that cardiovascular toxicity may be associated with the magnitude of the peak blood levels of cemadotin or its metabolites, whereas myelotoxicity depends on the duration of time that blood levels exceed a threshold concentration.

The first phase II clinical trial which used this drug at 2.5 mg/m^2^ daily 5-day schedule repeated every three weeks obtained clinical activity with durable response in chemotherapy-naïve patients with metastatic melanoma. Toxicity profile previously determined for this schedule was confirmed [117]. In contrast, no activity was observed with the same schedule in metastatic breast cancer patients previously treated with two lines of chemotherapy and in untreated non-small cell cancer patients [118, 119].

#### 2.3.4. Tasidotin

Tasidotin (ILX651) is a third-generation dolastatin 15 analogue that is metabolically stable through its resistance to hydrolysis [120]. It demonstrated* in vitro* cell cycle arrest in the G2 and M phases and inhibition of tubulin polymerization similar to cemadotin and the Vinca alkaloids. It can inhibit the extent of microtubule assembly even at low concentrations [121].* In vitro* study with MCF7/GFP breast cancer cells and* in vivo* pharmacokinetic analysis through LOX tumors xenografts proposed that tasidotin is converted in tasidotin C-carboxylate, a functionally active intracellular metabolite, 10 to 30 times more potent [122]. Capability of inducing apoptosis was observed in Ewing's sarcoma, rhabdomyosarcoma, synovial sarcoma, and osteosarcoma cell lines. Preclinical xenograft models of pediatric sarcomas showed antitumor activity [123].

Like cemadotin the schedule indicated for clinical use is daily administration for 5 days every 3 weeks. The recommended dose for investigation in phase II trial was 27.3 mg/m^2^/day. The toxicity profile was favourable and antitumor activity was found in melanoma patients [124]. The other two schedules were evaluated in phase I trial: 34.4 mg/m^2^ d1,3,5 q3 wk and 46.8 mg/m^2^ d1,8,15 q4 wk [125, 126]. Tolerability was similar with these schedules.

### 2.4. Rhizoxin

Rhizoxin (NSC 332598) is a macrolide antitumor antibiotic extracted from a pathogenic fungus,* Rhizopus chinensis*. It is known for its antifungal activity, but it is also studied for cytotoxic activity in a variety of human tumor cell lines, including melanoma, leukaemia, sarcoma, and some human tumor xenografts of melanoma, lung, and breast cancer [127]. The drug can bind to tubulin and inhibits microtubule assembly, blocking the cell cycle at the G2-M phase [128]. It is a more potent cytotoxic compound than vincristine* in vitro*, and, in addition, it showed activity in vincristine-resistant cells [129].

A recommended dose of 2.0 mg/m^2^ administered by i.v. bolus injection at 3-week intervals was identified through phase I trial because of its good tolerability with mucositis and neutropenia as the main toxicities [130]. Minimal or absent antitumor activity was found in phase II studies for patients with various advanced solid tumors [131–134]. A pharmacological study demonstrated the rapid and variable elimination of rhizoxin. These data could explain the low levels of systemic toxicity and the little response rates [135]. For this reason, alternative dosage and schedule were studied in phase I trial. A 72-hour continuous i.v. infusion indicated the dose of 1.2 mg/m^2^/72 hours as the MTD. The toxicity profile was similar to that obtained with brief infusion, but yet no antitumor responses were found [136].

### 2.5. D-24851

D-24851 (N-(pyridin-4-yl)-[1-(4-chlorbenzyl)-indol-3-yl]-glyoxyl-amid) is a synthetic compound which has been selected by a cell-based screening assay by ASTA Medica AG, Germany. This drug destabilizes microtubules by interacting with a binding site that does not overlap with those of known microtubule-destabilizing agents like vincristine or colchicine [137, 138].

D-24851 (also known as indibulin) induces Bcl-2 and Bax-mediated apoptosis in both p53^wt^ and p53^−/−^ cell lines [137, 139]. It produces* in vivo* curative effects in rat sarcomas at nontoxic doses, is suitable for oral use, does not give rise to neurotoxic effects at curative doses, unlike vincristine and paclitaxel, and is effective in MDR tumor cells, so that it is an excellent candidate as a chemotherapy agent [137]. In 2004, an LC/MS/MS (liquid chromatography/tandem mass spectrometry) system was proposed for quantitative analysis of D-24851 in human plasma and urine in phase I clinical trials. Indibulin was used in phase I/II clinical trials of patients with advanced solid tumors (metastatic breast cancer) [27, 140, 141]. In a phase I clinical trial indibulin was studied for oral administration once daily for 14 days every 3 weeks in patients with various solid tumors. Pharmacokinetic analysis showed a better tolerability under feeding condition. The recommended dose identified for further studies was 60 mg daily for 14 days. Dose-limiting toxicities were nausea and vomiting, which seemed to be related to solvent lactic acid [141].

Furthermore, the effects of two N-heterocyclic indolyl glyoxylamides derivatives of D-24851, BPR0C259, and BPR0C123 were investigated in NSCLC cells. The obtained results showed that these compounds can suppress the cell proliferation, by inducing p53-independent apoptosis and G2/M phase arrest, and potentially increase radiosensitivity of human lung cancer cells in a p53-independent manner [142].

### 2.6. Pseudolaric Acid B

Pseudolaric acid B (PAB) is a diterpene isolated from* Pseudolarix kaempferi Gordon* which is able to selectively inhibit the growth of actively proliferating cancer cells. It induces apoptosis through the intrinsic pathway, involving JNK/SAPK and p53. Nevertheless, its cytotoxic effects were found also in p53^−/−^ cell lines, which is interesting for its therapeutic use [143, 144].

It interacts with a different binding site on tubulin compared with those of colchicine and vinblastine [143] and, both* in vitro* and* in vivo,* inhibits endothelial cells proliferation and VEGF-dependent formation of blood vessels. In fact, PAB antagonizes VEGF-mediated antiapoptotic activity by inhibiting the phosphorylation/activation of KDR, the VEGF receptor implicated in mediating this effect [145]. Furthermore, at nontoxic doses, PAB inhibits VEGF secretion from tumor cells by reducing its HIF-1-dependent transcription. PAB, in fact, acts by accelerating the proteasome-mediated degradation of HIF-1*α*, by means of a mechanism so far unknown [146]. PAB also induces endothelial cell retraction, intercellular gap formation, and actin stress fiber formation, effects which can be attributed to disruption of tubulin cytoskeleton and which contribute to its antiangiogenic action [147]. Moreover, PAB circumvents P-glycoprotein overexpression-induced drug resistance and the doses used are well tolerated and nontoxic and have not proved lethal on tested animals [143]. PAB showed significant inhibitory effect and an additive inhibitory effect in combination with adriamycin on the growth of gastric cancer* in vivo* [148, 149].

### 2.7. Embellistatin

Embellistatin is a ketone isolated from* Embellisia chlamydospora* which inhibits microtubule polymerization and shows a strong antiangiogenic activity. It inhibits* in vitro* bovine endothelial cells (BAEC) proliferation through p53 and p21 activation, thus inhibiting bFGF-induced formation of vessels. This antiangiogenic activity has been confirmed* in vivo* on murine models. Similar effects have been found in human tumor cell lines, suggesting that it could be suitable for use in the development of new anticancer drugs [150].

### 2.8. CI-980

CI-980 (mivobulin) acts at the colchicine binding site and it appears to have significantly less vesicant activity than vinblastine [151]. It is a mitotic inhibitor with* in vivo* and* in vitro* activity against murine multidrug-resistant sublines. Its interactions with microtubules* in vitro* are similar to other drugs, but cellular microtubule and mitotic inhibition is more potent [152]. The uptake of CI-980 is not temperature or energy dependent, and its passive diffusion is followed by a significant but largely reversible binding to intracellular or membrane components [153].

Mivobulin was tested in a phase I trial using 24-hour infusion repeated every 3 weeks. MTD was 14.4 mg/m^2^. The main toxicities were neutropenia, dose-dependent but not dose-limiting, and early and reversible neurotoxicity characterized by dizziness, headache, loss of coordination, loss of consciousness, nervousness, and other symptoms. Tumor responses and tumor marker reductions were observed in a colon cancer patient and two ovarian cancer patients, respectively [154]. The same toxicity profile was confirmed in other studies [155, 156]. A continuous 72-hour infusion of MTD 4.5 mg/m^2^/day every 21 days was associated with reduced neurotoxicity but dose-limiting neutropenia [157]. For this reason, it was used in phase II clinical trials. A similar tolerability profile was found. CI-980 seems inactive in metastatic colorectal carcinoma, advanced soft-tissue sarcomas, treated and untreated melanoma, hormone-refractory prostate cancer, and malignant gliomas [158, 159]. Minimal activity was observed in platinum-refractory advanced epithelial ovarian carcinoma [160].

### 2.9. T138067

T138067 is a synthetic compound which irreversibly disrupts microtubule assembly by a selective and covalent binding to beta1, beta2, and beta4 isotypes of beta-tubulin at a conserved cysteine residue (Cys-239). Its action results in cell cycle arrest at G2/M and induction of apoptosis. It exhibits cytotoxic activity in tumor cell lines resistant to various antimicrotubule agents (vinblastine, paclitaxel, etc.) and in multidrug-resistant human tumor xenografts [161]. The covalent interaction of T138067 with *β*-tubulin may be proposed as a new way to overcome MDR.* In vivo* studies showed that this drug can cross the blood-brain barrier in mice, suggesting a possible use for brain tumors [162].

Phase I trials of T138067 were conducted by using a 3-hour infusion of drug given weekly or every 21 days with a recommended dose of 330 mg/m^2^ per week. DLTs were neutropenia and neurological effects, consisting of encephalopathy, headache, hearing loss, and ataxia [163, 164]. This weekly dosage was used in two phase II clinical trials for patients with malignant glioma and metastatic colorectal cancer previously treated with irinotecan and 5-fluorouracil, respectively. The good toxicity profile was confirmed in both studies. No clinical activity in terms of antitumor responses was observed in both cases [165, 166].

#### 2.9.1. T900607

T900607 is similar to T138067 for the kind of binding to tubulin in Cys-239 residue, but it is distinguished for a reduced ability to cross the blood-brain barrier.

A phase I trial indicated a recommended dose of 130 mg/m^2^ delivered in i.v. infusion over 60 minutes on a 21-day cycle. No objective responses were observed but stable disease was reported in 7/20. Cardiac toxicity is the main drug-related side effect with this schedule. A different schedule consisting of weekly administration of T900607 identified MTD of 100 mg/m^2^. This schedule was used in a phase II clinical trial for untreated patients with unresectable hepatocellular carcinoma. It showed good tolerability and moderate activity in some of these patients [167].

### 2.10. ABT-751

ABT-751, also known as E7010, is a sulfonamide able to impair microtubule formation and inhibit cell growth. Its binding characteristics seem to be different from that of colchicine and Vinca alkaloids. This agent has antiproliferative effects in many tumor cell lines which are drug-resistant due to the P-glycoprotein overexpression [168]. It showed a broad spectrum of activity against a variety of tumors in mice and human tumor xenografts, when administered orally [169]. Beta3 isotype is the preferential binding target. ABT-751-resistant cells were characterized by decreased expression of this tubulin isotype [170]. A warning derived from an* in vivo* study, which shed light on a possible testicular toxicity related to this drug administration in mice. It consisted of loss of seminiferous epithelial cells due to apoptosis of meiotic spermatocytes [171]. This drug selectively reduces tumor blood flow through tumor necrosis, regardless of a direct cytotoxic effect on tumor cells. Negligible is the effect on normal vascular function [172].

In a phase I clinical trial ABT-751 was administered as oral single or 5-day doses. The recommended dose for phase II trials is identified at 320 mg/m^2^ for single dose and 200 mg/m^2^/day for 5-day repeated dose. Peripheral neuropathy and intestinal paralysis were the DLTs. Gastrointestinal toxicity was dose-dependent but hematological toxicity was not dose-dependent [173]. Pharmacokinetic analysis of this study suggested that activity of ABT-751 may be time-dependent. For this reason a new schedule using a divided dose in order to maintain the blood level of ABT-751 has been formulated. The recommended dose in hematologic malignancies is 175 mg/m^2^/day orally for 21 days every 4 weeks [174]. In a phase I trial for a pediatric population of patients with solid tumors ABT-751 was administered orally once daily for 21 days, repeated every 28 days. The MTD obtained for this schedule was 100 mg/m^2^/day. DLTs included fatigue, sensory neuropathy, transient hypertension, neutropenia, thrombocytopenia, nausea, vomiting, dehydration, abdominal pain, and constipation [175]. In a phase II clinical trial, 21-day every 28 days schedule at the dose of 200 mg daily was studied in taxane-refractory NSCLC patients. Toxicity was acceptable. Median time to tumor progression and overall survival was 2.1 and 8.4 months, respectively. The objective response rate was 2.9% [176]. The combination of this agent with other cytotoxic drugs was proposed for future clinical studies. A phase IB study investigated clinical antitumor activity of ABT-751 in combination with docetaxel in patients with castration-resistant prostate cancer. Based on the cumulative safety analysis, the recommended phase II dose of ABT-751 is 200 mg daily with docetaxel 60 mg/m^2^ for this patient population [177]. Further phases I and II clinical trials were carried out to evaluate activity of ABT-751 in combination with other drugs in advanced or metastatic NSCLC patients [178, 179]. ABT-751 showed adverse effects, although it has the advantage of being orally bioavailable.

## 3. Microtubule-Stabilizing Agents

**Figure 5:**
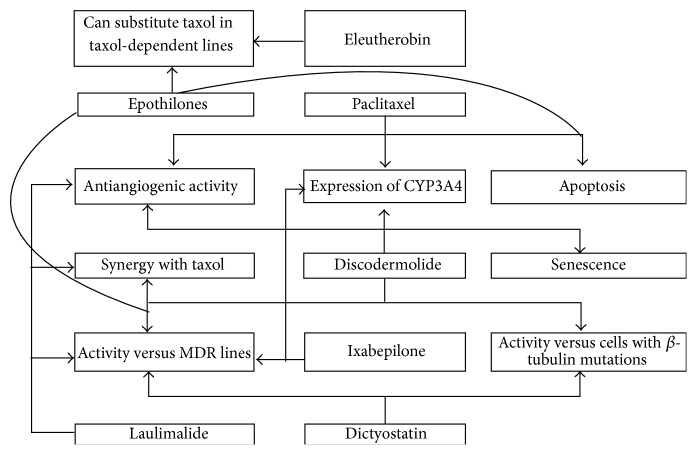
Similarities and differences between mechanisms of action and activity of microtubule-stabilizing agents.

Unlike the microtubule-destabilizing agents, there are other compounds that enhance microtubule polymerization. One of the most important classes of microtubule-stabilizing chemotherapy agents is that of taxanes, which target the cytoskeleton and spindle apparatus of tumor cells by binding to the microtubules, thereby disrupting key cellular mechanisms, including mitosis. The first microtubule-stabilizing agent used in anticancer chemotherapy [180] was paclitaxel. Thanks to their peculiar mechanism of action, taxanes are among the most effective chemotherapeutic agents used in the treatment of multiple solid tumors, such as breast, ovarian, lung, and prostate cancers. However, the occurrence of resistance limits treatment options and creates a major challenge for clinicians. Several potential mechanisms of resistance to these drugs have been identified, occurring at different pharmacodynamics levels. Besides the well-known overexpression of Pgp, an ABC transmembrane transporter which pumps the drugs out of the tumor cells [8], the alterated expression of specific beta-tubulin isotypes, seems to play an important role. Most notably, the increased expression of *β*III-tubulin isotype has been associated with resistance to taxanes in several cancers, including ovarian, breast, and lung cancer [9, 181, 182]. It was originally correlated to the qualitative or quantitative modifications of the microtubule complex, which represents the target of such agents, definitively reducing the drug binding affinity [27]. However, the aberrant expression of *β*III-tubulin can also interfere with microtubule dynamics, increasing the dynamic instability and counteracting the stabilizing effect of taxanes, with consequences for drug sensitivity/resistance [183]. Recent studies have suggested *β*III-tubulin as a prosurvival factor adaptively expressed by cancer cells exposed to microenvironmental stressors, such as hypoxia or deficient nutrient supply [184, 185]. The activation of the *β*III-tubulin-dependent pathway in partnership with GTPases, such as guanylate-binding protein 1 (GBP1), is associated with the incorporation of PIM1 into the cytoskeleton of tumor cells, conferring a survival advantage in a hostile microenvironment and ultimately leading to the development of drug resistance [186]. Finally, a multitude of alterations involving the apoptotic signaling pathways downstream the microtubule complex, as well as aberrant expression of microRNA, have been also found in resistant tumors. A better understanding of the mechanism underlying the occurrence of acquired resistance has led to the development of a new class of microtubule-stabilizing agents, including epothilones, discodermolide, sarcodictyins, eleutherobin, and laulimalide, which are more readily modifiable, with different structures but a similar mechanism of action [187] (Table 2). Epothilones, discodermolide, eleutherobins, and sarcodictyins compete with paclitaxel for binding to microtubules and bind at or near the taxane site, whereas laulimalide seems to bind to unique sites on microtubules (Figure 5). Recently, a novel generation of paclitaxel derivatives have been designed, targeting a specific intermediate binding site in the microtubule with differential affinity, depending on the *β*-tubulin isotype expressed in the tumor. Since *β*III-tubulin is overexpressed in the majority of aggressive, resistant tumors, the design of a *β*III-tubulin targeted agent was expected to enhance the drug activity, reducing common toxicities. However, none of the new molecules tested in breast cancer cell lines was superior to the currently used taxanes [188].

### 3.1. Epothilones

Among several classes of microtubule-targeting chemotherapy agents that may maintain activity despite clinical resistance to taxanes, there are the epothilones which have been isolated from the soil bacterium* Solangium cellulosum* and have been studied most extensively in the clinical setting [189]. They induce the formation of an aberrant mitotic spindle, mitotic arrest, and apoptosis [190]. Their greater solubility in water and their activity in MDR cells have made them an alternative to paclitaxel in anticancer treatment [191, 192]. Moreover, their simple structure makes it easy to produce synthetic analogs during the clinical experimentation phase [190]. There are 4 classes of natural epothilones (A, B, C, and D). By means of the selection of resistant or taxane-dependent cells, it has been observed that tubulin *β*1 plays an important role in epothilone B functionality [193].

Ixabepilone (Ixempra) is a semisynthetic analog of epothilone B, selected because of its greater metabolic stability and its simple preparation. It is more powerful* in vitro* than paclitaxel and also presents cytotoxicity against MDR cells. It causes regression of MDR tumors and is more effective than paclitaxel in a wide spectrum of pediatric tumors [194]. Ixabepilone is currently the only approved epothilone derivative and the most clinically advanced (phases II and III clinical trials), showing efficacy in several patient subgroups and in various stages of breast cancer. This analog is used for the treatment of locally advanced or metastatic breast cancer as monotherapy after failure of a taxane, an anthracycline, and capecitabine, or in combination with capecitabine after failure of a taxane and an anthracycline [195].

A great number of phase II clinical studies of epothilones in cancer treatment have been reported, and significant activity in taxane-sensitive tumor types (such as breast, lung, and prostate cancers) has been observed [12, 17]. Response rates in taxane-refractory metastatic breast cancer are relatively modest, but ixabepilone and patupilone have shown promising efficacy in hormone-refractory metastatic prostate cancer and in taxane-refractory ovarian cancer [196, 197].

### 3.2. Laulimalide

Laulimalide is a macrolide isolated from marine sponge (*Cacospongia mycofijiensis*) which inhibits cell proliferation, promoting assembly of the microtubules and stabilizing them in a taxol-like way, but at a different binding site located on two adjacent *β*-tubulin units between tubulin protofilaments of a microtubule [198–200]. This agent is also active in MDR cells which overexpress glycoprotein-P. When administered below cytotoxic doses, the drug prevents blood vessel formation and the VEGF-induced endothelial cell migration [201]. Docetaxel and laulimalide possess a synergic effect in these two processes, whereas they have antagonistic effects towards cell proliferation.

Used at low doses, laulimalide inhibits events downstream of VEGFR-2 activation, such as FAK and paxillin phosphorylation, VEGFR-2/FAK/Hsp90 interaction, and integrin activation. Compared with docetaxel, laulimalide has less effect on the VEGF-induced VEGFR-2/integrin *α*5*β*1 interaction and is more effective with regard to phosphorylated paxillin levels. Furthermore, it inhibits RhoA/integrin *α*5*β*1 association, suggesting that synergic effects of the two drugs might be explained by two different modalities of action.

The low quantities of the drug found in nature, together with its instability caused by its transformation into isolaulimalide, have led to the synthesis of the drug itself and of several analogs. The removal of a electrophilic and/or nucleophilic group, which prevents the substitution process, leads to major functional stability of the drug [202]. In preclinical phase, laulimalide so far showed poor efficacy and systematic toxicity [12]. The macrolide peloruside A shared many of the same properties of laulimalide, including its binding site and synergistic effects with the taxanes [203].

### 3.3. Dictyostatin

Dictyostatin is a macrolactone produced from sponges which induces* in vitro* tubulin assembly in the same way of paclitaxel but more rapidly. Like discodermolide, this drug possesses an antiproliferative action against paclitaxel-resistant human tumor cells as a result of *β*-tubulin mutations [204]. Dictyostatin inhibits the binding of discodermolide with microtubules and both drugs are able to inhibit the binding of epothilone B and paclitaxel with microtubules [204]. Several discodermolide/dictyostatin hybrids have been designed and have been found to maintain antiproliferative activities against several taxane-resistant cell lines [205, 206].

### 3.4. Eleutherobin

Eleutherobin is a glycosylate diterpene isolated from* Eleutherobia sp.* [207], which inhibits cell proliferation stabilizing microtubules. It binds at a site overlapping that of paclitaxel [208]. There is another group of cytotoxic agents, called sarcodictyins, which are structurally and functionally correlated to eleutherobins but not so toxic [209]. A form of cytotoxic diterpene, known as (Z)-sarcodictyine A, has been isolated from* Bellonia albiflora*; this exhibits a high level of toxicity towards human HeLa cells of the cervix [210]. Eleutherobin and the sarcodictyins have not been pursued clinically likely due to their susceptibility to Pgp-mediated transport [211].

## 4. Clinical Implications

In the last few years, a great amount of efforts has been put into the identification of new microtubule-targeting agents for use in anticancer therapy [212]. These last generation agents are also active in MDR tumors, which are resistant to the traditional antitubulin drugs used in chemotherapy, such as Vinca alkaloids and taxanes. Furthermore, these compounds have shown significant antivascular and antiangiogenic activity, leading to the possibility of using them both as alternatives to or in combination with preexistent drugs, as already indicated in several published studies [213]. A lot of clinical trials were conducted to study microtubule-targeting agents. In particular, epothilones are in advanced phases of clinical development [214, 215]. In cancer therapy, microtubule-targeting agents can target angiogenesis, cell migration, and intracellular trafficking to prevent tumor growth and induce cancer cell apoptosis. These new agents, which impair or enhance tubulin polymerization, can be classified in two groups: natural and synthetic drugs. The natural compounds are derived from different species of uni- and multicellular organisms. To improve their pharmacodynamic and pharmacokinetic features some of these compounds are transformed in semisynthetic molecules. Other agents are produced by a totally synthetic procedure. The great diversity of natural and synthetic compounds capable of interacting with microtubules represents an important source for developing of novel potential anticancer agents. However, the effectiveness of these agents in cancer therapy has been impaired by various side effects and drug resistance. Phase I trials have allowed identifying more tolerable schedules with the most frequent toxicities represented by neutropenia and neurological, cardiovascular, and gastrointestinal effects. The main way of delivery is the i.v. infusion. Oral assumption was studied for the synthetic compounds D-24851 and ABT-751. The most evident efficacy was observed for rhizoxin, above all in NSCLC. For the other agents only minor or no responses were obtained. The identification of new schedules or the transformation in more potent analogues should allow overcoming these hurdles in their clinical advancement.

## 5. Conclusions

Data obtained up till now have allowed introducing some of these microtubule-targeting drugs into the clinical experimentation phase, whereas others, still in their preclinical phase, represent excellent candidates for a future use in cancer treatment, thus opening new roads towards the development of new, individual, and efficient therapeutic approaches.

## Figures and Tables

**Table 1:** Microtubule-destabilizing agents.

Chemical lead	Properties and effects	Clinical trial/status	References
Cryptophycins	Apoptosis induction. Synergistic with chemotherapy and radiation.	Phase II clinical trials in platinum-resistant ovarian cancer and in NSCLC (C-52) but withdrawn due to peripheral neuropathy.	[26, 28, 31, 32, 36]

Combretastatin A-4-P	Antivascular and antiangiogenic activity. Synergistic with radiation, hyperthermia, chemotherapy, and immunoradiotherapy.	Phases II and III clinical trials in advanced solid tumors (lung and thyroid cancer) and in combination with carboplatin.	[63, 64, 66–70, 72, 73]

Combretastatin A-1-P	Antivascular and antitumoral activity superior to CA-4-P. Synergistic with chemotherapy.	Phase I clinical trials in solid tumors and in acute myelogenous leukaemia and myelodysplastic syndromes.	[78, 79]

Ombrabulin	Antivascular and antitumoral activity superior to CA-4-P. Synergistic with chemotherapy.	Phase I clinical trials as a single agent or in combination; phase III clinical trial in advanced soft-tissue sarcoma.	[86]

Soblidotin	Apoptosis induction. Antivascular activity. Antitumoral activity in tumors resistant to vincristine, docetaxel, and paclitaxel.	Phase II clinical trials in advanced solid tumors (soft-tissue sarcoma, NSCLC).	[99–105]

D-24851	Curative at nontoxic doses in rat tumor. No neurotoxic effects. Oral applicability. Activity versus MDR cell lines.	Phase I/II clinical trials in advanced solid tumors.	[140, 141]

Pseudolaric acid B	Antiangiogenic activity. No neurotoxic effects in tested animal. Activity versus MDR cell lines.	Preclinical phase.	[148, 149]

Embellistatin	Antiangiogenic activity.	Preclinical phase.	[150]

**Table 2:** Microtube-stabilizing agents.

Chemical lead	Properties and effects	Clinical trial/status	References
Epothilones	Elevated water solubility, activity versus MDR cell lines, and chemical malleability.	Phase II/III clinical trials in taxane-sensitive solid tumors (breast, lung, and prostate).	[195, 196]

Ixabepilone	Epothilone B analog, superior metabolic stability, and activity versus MDR cell lines.	Approved in 2007 for metastatic breast cancer; several ongoing trials in solid tumors.	[194, 197]

Laulimalide	Activity versus MDR cell lines and angiogenic activity, synergistic with docetaxel.	Preclinical phase.	[199, 201]

Dictyostatin	Activity against MDR cell lines, synergistic with taxol.	Preclinical phase.	[205, 206]
